# Analysis of distinct long noncoding RNA transcriptional fingerprints in pancreatic ductal adenocarcinoma

**DOI:** 10.1002/cam4.1027

**Published:** 2017-02-21

**Authors:** Xiang Yu, Yang Lin, Wu Sui, Yanfen Zou, Zhongchuan Lv

**Affiliations:** ^1^ Department of General SurgeryThe Affiliated Yantai Yuhuangding Hospital of Qingdao UniversityYantaiChina; ^2^ Department of Obstetrics and gynecologyThe Affiliated Yantai Yuhuangding Hospital of Qingdao UniversityYantaiChina

**Keywords:** Biomarker, CASC9, LINC00152, lncRNA, PDAC

## Abstract

Pancreatic ductal adenocarcinoma (PDAC) is one of the most aggressive and lethal malignancies with the worst prognosis. Recent studies have demonstrated that long noncoding RNAs (lncRNAs) play critical roles in tumorigenesis and cancer progression. However, the expression pattern and roles of lncRNAs in the development of PDAC remain unknown. Herein, we globally analyzed the lncRNA expression profile in human PDAC and non‐tumor tissues using four independent public microarray datasets from Gene Expression Omnibus (GEO). The analysis of GEO datasets by repurposing microarray probes confirmed that hundreds of lncRNAs are differentially expressed in PDAC tissues compared with normal tissues. We selected four lncRNAs including LINC00152, CASC9, LINC00226 and F11‐AS1 for validation in PDAC cell lines and normal cells. Loss of function assays were performed to investigate the roles of LINC00152 and CASC9 in PDAC cell proliferation and invasion. Taken together, our findings demonstrate lncRNA expression alterations in PDAC and may provide new potential molecular markers for PDAC patient diagnosis and treatment.

## Introduction

Pancreatic ductal adenocarcinoma (PDAC) is a common digestive system cancer and one of the most aggressive malignancies with a very poor outcome 1, 2. Despite substantial efforts to uncover the molecular and genetic basis of this disease, the 5‐year survival rate has remained <5% due to metastasis and resistance to chemotherapy and radiation therapy 3. Hence, there is an urgent need for understanding of the mechanisms of PDAC development and the identification of effective markers for early diagnosis and drug targets of PDAC. Over the past decades, most research of molecular markers for PDAC have focused on proteins and microRNAs, such as K‐Ras 4, miR‐221 5, and recently studies hotspot the long noncoding RNAs (lncRNAs).

Recently, the advance on the high‐throughput sequencing technique and bioinformatics has led to the complement of the whole human genome sequencing and The Encyclopedia of DNA Elements (ENCODE) project 6, 7. These sequencing data revealed that <2% of the human genome are protein coding genes, while the majority of the rest of the genome contains noncoding genes, yielding thousands of transcripts without protein coding ability 8. LncRNAs are newly identified non‐coding RNAs >200 nt in length that lack protein coding capacity 9, 10. Many studies have demonstrated that lncRNAs participate in many cellular processes, such as X chromatin imprinting, chromatin remodeling, alternative splicing, cell fate decision, cell differentiation, immune response, cancer cell metastasis, and drug resistance 11, 12, 13. Increasing evidence has linked lncRNA alterations with multiple human diseases, particularly cancers. For example, the lncRNA TUG1 enhances tumor‐induced angiogenesis and VEGF expression through inhibiting miR‐299 in human glioblastoma 14, and lncRNA SLNCR1 mediates melanoma invasion through binding with brain‐specific homeobox protein 3a[Abbreviations only used once in the manuscript have been removed.] and the androgen receptor at a conserved SRA1‐like region, and thereby transcriptional activation of matrix metalloproteinase 9 15.

Several studies have documented the role of lncRNAs in PDAC development and progression. For example, increased AFAP1‐AS1 expression was associated with PDAC patient poor survival and short‐term recurrence 16, and the lncRNA MALAT1 promoted aggressive pancreatic cancer proliferation and metastasis via HuR‐TIA‐1 mediated autophagic activation 17. Moreover, the lncRNA MIR31HG exhibits oncogenic properties and promotes PDAC progression by acting as an endogenous ‘sponge’ through competing for miR‐193b binding to regulate its target 18. Although some of these lncRNAs have been characterized in PDAC, the expression pattern and roles of the majority of lncRNAs in the development and progression of PDAC remain unknown. To evaluate the lncRNA expression profile and identify the PDAC associated lncRNAs, we examined differential expression profiles of lncRNAs in PDAC samples and adjacent non‐tumor samples by analyzing four microarray gene profiling datasets from Gene Expression Omnibus (GEO). This study uncovers the aberrant lncRNAs expression profiling in PDAC tissues and cells, which may provide new potential molecular markers for PDAC diagnosis and treatment.

## Methods and Material

### Public microarray datasets analysis

Five public microarray datasets (GSE15471, GSE28735, GSE62165, GSE62452, and GSE40098) were retrieved from the publically available database GEO. LncRNA expression profiling of GEO datasets was analyzed using the Affymetrix Human Genome U133 Plus 2.0 Array platform, Gene 1.0 ST Array (Affymetrix, Santa Clara, CA) and Agilent‐014850 Whole Human Genome Microarray 4 × 44K G4112F. The recent release of the HG‐U133Plus2 array includes 6492 probe sets corresponding to 5563 lncRNAs. R software and packages were used to preprocess RNA sequencing and microarray data.

### Cell culture and siRNA transfection

SW1990 and BxPC‐3 cells were purchased from the Shanghai Cell Bank of the Chinese Academy of Sciences (Shanghai, China). BxPC‐3 cells were cultured in RPMI 1640 (Invitrogen, Shanghai, China), and SW1990 cells were cultured in Dulbecco's modified Eagle's medium. Both cell cultures were supplemented with 10% fetal bovine serum and 1% penicillin and streptomycin (Invitrogen). The LINC00152, CASC9 and negative control siRNAs were transfected into SW1990 or BxPC‐3 cells using Lipofectamine 2000 (Invitrogen) according to the manufacturer's instructions. At 48 h post‐transfection, cells were harvested for RNA extraction and qPCR analysis. The siRNA sequences are listed in Table S2

### RNA extraction and qRT‐PCR

RNA was extracted from PDAC cells using TRIZOL reagent, according to the manufacturer's instructions. RNA (1 *μ*g) was reverse transcribed into cDNA using oligo dT and random primers and the PrimeScript RT Reagent Kit (TaKaRa, Dalian, China), according to the manufacturer's instructions. To examine lncRNA expression levels, SYBR Premix Ex Taq (TaKaRa) was used following the manufacturer's instructions. The housekeeping gene Actin Beta (ACTB) was used as an internal control. The primer sequences of lncRNAs are listed in Table S2 qRT‐PCR analysis was performed on ABI7500, and data were calculated using the comparative cycle threshold (CT) (2^−ΔΔCT^) method.

### Cell proliferation assay

The Cell Proliferation Reagent Kit I (MTT) (Roche Applied Science) was used for assays. SW1990 and BxPC‐3 cells were seeded into 96‐well plates. Then, 20 *μ*L MTT solution were added into each well, and plates were incubated for 6 h. Finally, the absorbance at 490 nm was measured.

### Transwell assays

Transwell assays (Corning, Tewksbury, MA 8.0‐*μ*m pores) were used to measure PDAC cell invasion ability after transfection with LINC00152, CASC9 or negative control siRNAs. Cells (1 x 10^5^) in 1% FBS containing medium were placed into the upper chamber of an insert coated with Matrigel (Sigma‐Aldrich). Medium containing 10% FBS was added to the lower chamber. After incubation for 24 h, the cells that had invaded through the membrane were stained with methanol and 0.1% crystal violet, imaged, and counted using an IX71 inverted microscope (Olympus, Tokyo, Japan).

### Statistical analysis

The one‐way ANOVA, Mann**–**Whitney *U* test, and Students *t* test (2 tailed) were conducted to analyze in vitro data using R software and Bio‐conductor. *P* < 0.05 were considered statistically significant.

## Results

### Identification of lncRNAs profiling in PDAC tissues

**Figure 1:**
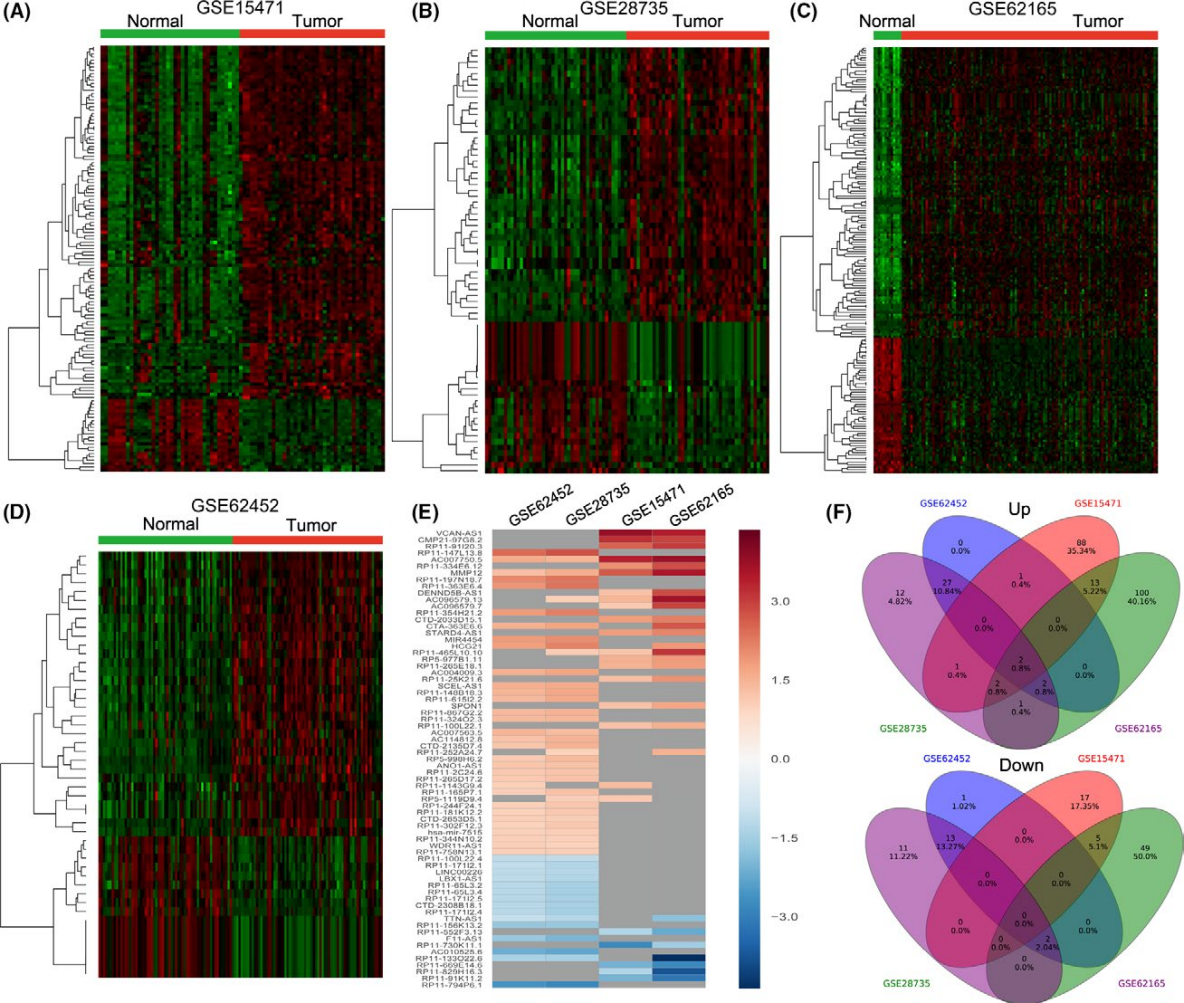
The expression pattern of lncRNAs in human PDAC and normal tissues. (A) Heatmap of the altered lncRNAs expression profiles in PDAC and parental normal tissues were analyzed using the GSE15471 datasets. (B) Heatmap of the altered lncRNAs expression profiles in PDAC and parental normal tissues were analyzed using the GSE28735 datasets. (C) Heatmap of the altered lncRNAs expression profiles in PDAC and parental normal tissues were analyzed using the GSE62165 datasets. (D) Heatmap of the altered lncRNAs expression profiles in PDAC and parental normal tissues were analyzed using the GSE62452 datasets. (E) Heatmap of the altered lncRNAs expression profiles (consistently altered at least two datasets, fold change) in four datasets. (F) Venn diagram of differentially expressed lncRNAs in four datasets.

To examine the lncRNA expression profile in PDAC tissues, we used the PDAC and non‐tumor microarray gene profiling datasets (GSE15471, GSE28735, GSE62165, GSE62452) from GEO. The GSE15471 dataset consists of 36 paired normal‐tumor samples; GSE28735 consists of 45 matching pairs of pancreatic tumor and adjacent non‐tumor tissues; GSE62165 consists of 118 PDAC samples and 13 control samples; and GSE62452 consists of 69 pancreatic tumors and 61 adjacent non‐tumor tissue. Analysis of these datasets showed that 129 lncRNAs expression were dysregulated in the GSE15741 dataset (107 upregulated and 22 downregulated); 73 lncRNAs was differentially expressed in the GSE28735 dataset (47 upregulated and 26 downregulated); 176 lncRNAs were dysregulated in the GSE62165 dataset (120 upregulated and 56 downregulated); and 48 lncRNAs were differentially expressed in the GSE62452 dataset (32 upregulated and 16 downregulated) (Fig. 1A–D, Table S1). Further analysis showed that 69 lncRNAs were upregulated or downregulated at least two datasets (Fig. 1E and F). These data indicate that many lncRNAs are differently expressed in PDAC and that these could be candidate biomarkers for PDAC diagnosis.

### Validation of lncRNA expression in PDAC and normal cell lines

**Figure 2:**
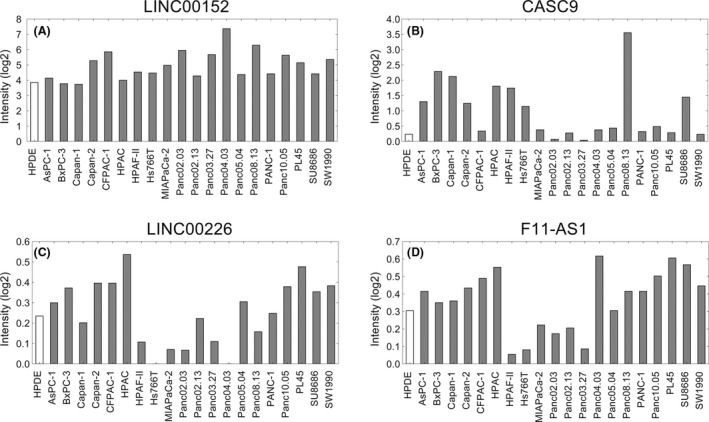
Elevate the lncRNAs expression in PDAC cell lines and normal cells. (A, B) The expression levels of LINC00152 and CASC9 were detected in 20 PDAC cell lines and an immortalized non‐malignant pancreatic duct cell line (HPDE) using GSE40098 dataset. (C, D) The expression levels of LINC00226 and F11‐AS1 were detected in 20 PDAC cell lines and an immortalized non‐malignant pancreatic duct cell line (HPDE) using GSE40098 dataset.

To validate these lncRNAs in PDAC cell lines, we used the PDAC cell line microarray gene profiling data (GSE40098) from GEO. We selected two upregulated lncRNAs (LINC00152 and CASC9) that were reported to be upregulated in other cancers, and two mostly downregulated lncRNAs (LINC00226 and F11‐AS1) for further validation in PDAC cell lines and the normal cell line HPDE. CASC9 and LINC00152 were overexpressed in most of the PDAC cell lines compared with HPDE cells (Fig. 2A and B), while LINC00226 expression was downregulated in 10 cell lines compared with HPDE cells and F11‐AS1 was only downregulated in 6 cell lines compared with HPDE cells (Fig. 2C and D).

### Knockdown of LINC00152 and CASC9 impaired PDAC cells proliferation

**Figure 3:**
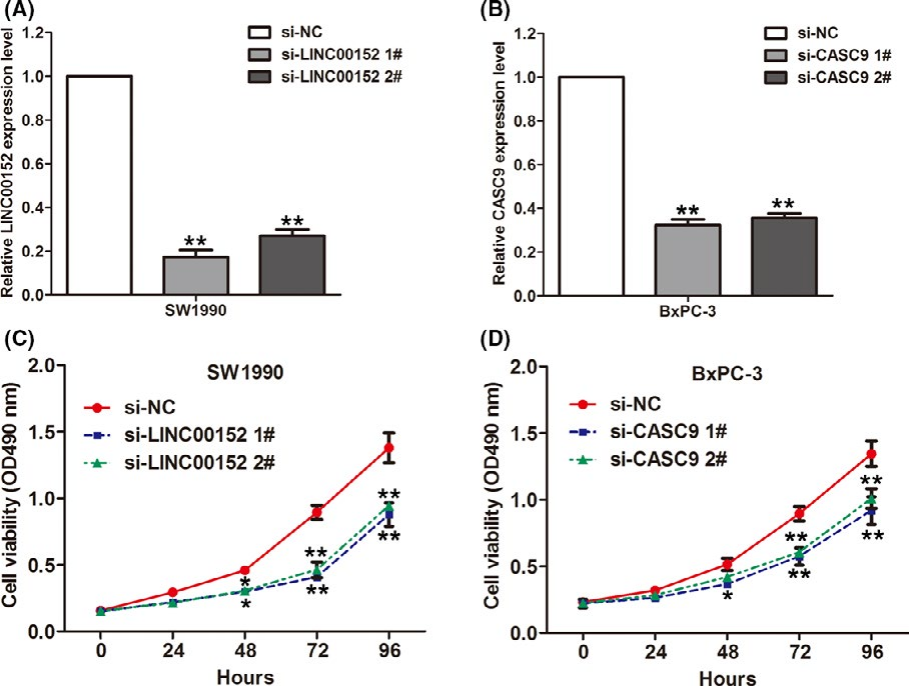
Knockdown of LINC00152 and CASC9 inhibits PDAC cells proliferation. (A) The expression levels of LINC00152 were detected by qRT‐PCR in SW1990 cells after transfection with LINC00152 siRNAs, or negative siRNA. (B) The expression levels of CASC9 were detected by qRT‐PCR in BxPC‐3 cells after transfection with CASC9 siRNAs, or negative siRNA. (C) MTT assay was performed to determine the proliferation of si‐LINC00152 or si‐NC transfected SW1990 cells. Data represent the mean ± S.D. from three independent experiments. (D) MTT assay was performed to determine the proliferation of si‐CASC9 or si‐NC transfected SW1990 cells. Data represent the mean ± S.D. from three independent experiments. **P <* *0.01; *P < 0.05

To investigate whether these altered lncRNAs are involved in PDAC development, we chose upregulated lncRNAs LINC00152 and CASC9 for further study. LINC00152 was overexpressed in multiple cancers including gastric cancer 19, hepatocellular carcinoma 20, and gallbladder cancer 21 et al. Increased LINC00152 could promote cancer cell proliferation, invasion, and metastasis. Moreover, a recent study showed that CASC9 expression is upregulated and regulates cell migration and invasion in esophageal cancer 22. We designed siRNAs for LINC00152 and CASC9 and transfected them into SW1990 or BxPC‐3 cells. The qRT‐PCR results confirmed significantly reduced expression of these lncRNAs in siRNA‐transfected cells compared with controls (Fig. 3A and B). MTT assays showed that knockdown of LINC00152 could significantly impair SW1990 cell proliferation (Fig. 3C), while knockdown of CASC9 could significantly impair BxPC‐3 cell proliferation (Fig. 3D). These data indicated that these lncRNAs might play important roles in PDAC development.

### Knockdown of LINC00152 and CASC9 inhibits PDAC cell invasion

**Figure 4:**
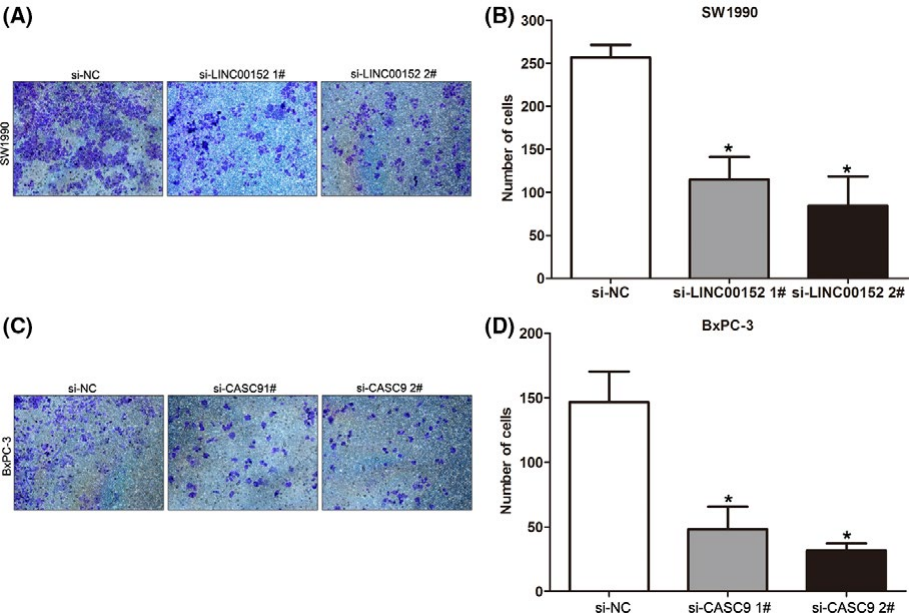
Knockdown of LINC00152 and CASC9 inhibits PDAC cells invasion. (A, B) Transwell assays was performed to determine the invasive ability of si‐LINC00152 or si‐NC transfected SW1990 cells. Data represent the mean ± S.D. from three independent experiments. (C, D) Transwell assays was performed to determine the invasive ability of si‐CASC9 or si‐NC transfected BxPC‐3 cells. Data represent the mean ± S.D. from three independent experiments. *P < 0.05. PDAC, Pancreatic ductal adenocarcinoma

Cancer cell invasion and metastasis is a significant aspect of PDAC progression, and may lead to poor prognosis and short survival for PDAC patients. To further investigate the potential biological function of LINC00152 and CASC9 in PDAC progression, we performed transwell assays to examine the effect of LINC00152 and CASC9 on PDAC cell migration and invasion. LINC00152 downregulation significantly impeded SW1990 cell invasion compared with controls (Fig. 4A and B). Similarly, knockdown of CASC9 significantly impaired BxPC‐3 cell invasion compared with controls (Fig. 4C and D). These results suggest that LINC00152 and CASC9 have oncogenic functions that could promote PDAC cell invasion.

## Discussion

Over the past decade, increasing studies have highlighted the important roles of human genome non‐coding elements in human diseases, specifically cancer development and progression 13, 23, 24. LncRNAs have been reported to contribute to the development and progression of multiple human cancer by functioning as oncogenes or tumor suppressors through the activation or repression of target expression 25, 26. Importantly, several studies showed that lncRNAs expressions are tissue‐specific, and several certain cancer‐specific lncRNAs have been characterized. For example, Sun et al. found that the gastric cancer‐associated lncRNA HOXA11‐AS is specifically upregulated in human gastric cancer tissues, but not in other cancer tissues 27. Moreover, Xiang et al. reported that lncRNA CCAT1‐L is transcribed specifically in human colorectal cancers from a locus 515 kb upstream of MYC 28. These findings suggest that lncRNAs may be used as biomarkers for human different cancers. However, the PDAC‐associated lncRNAs are still not well‐documented.

In this study, we globally assessed lncRNA expression patterns in PDAC tissues compared with their parental normal tissues or non‐tumor tissues using microarray gene profiling datasets from GEO. Our results showed that hundreds of lncRNA were differentially expressed in PDAC tissues compared with normal tissues or non‐tumor tissues. We selected the upregulated lncRNAs LINC00152 and CASC9 and downregulated lncRNAs LINC00226 and F11‐AS1 and validated expressions in PDAC cell lines and normal cell line HPDE using microarray gene profiling data from GEO. CASC9 and LINC00152 were upregulated in most of the PDAC cell lines, while LINC00226 and F11‐AS were downregulated in half of the PDAC cell lines. These results suggest that mis‐regulated lncRNAs in PDAC might play important roles in PDAC development and progression.

A recent study reported that upregulation of LINC00152 by the SP1 transcription factor promotes gallbladder cancer cell growth and tumor metastasis by affecting the PI3K/AKT pathway 21. In addition, Yue et al. found that LINC00152 could function as a competing endogenous RNA to modulate the expression of miR‐193a‐3p, and then confer oxaliplatin resistance; LINC00152 may be used as a candidate prognostic indicator of outcome and drug responsiveness in colon cancer 29. Moreover, the lncRNA CASC9 was found to be markedly upregulated in esophageal squamous cell carcinoma tissues, and knockdown of CASC9 significantly suppressed esophageal squamous cell carcinoma cell migration and invasion 22. However, there is no evidence about the expression pattern and role of LINC00226 and F11‐AS1 in human cancers. Furthermore, our results found that knockdown of CASC9 and LINC00152 impaired PDAC cell proliferation and invasion compared with control cells. These data suggest that the mis‐regulated lncRNAs such as LINC00152 and CASC9 play important roles in PDAC development and progression.

In summary, our findings showed that hundreds of lncRNAs were differently expressed in PDAC tissues and cell lines compared with their parental normal tissues or cells. Some of these lncRNAs might play critical roles in PDAC development and progression through regulation of target protein target genes and their underlying pathways. Our study highlights the important roles of lncRNAs in PDAC and provides candidates as diagnostic markers and potential targets for PDAC. The present study also has a few limitations; for example, many lncRNAs might have been missed in this study. This will need to be further investigated using RNA sequencing data.

## Conflicts of Interest

No potential conflicts of interest were disclosed.

## Supporting information


**Table S1.** Differently expressed lncRNAs in four GEO data sets.

**Table S2.** Sequences for primers and siRNAs.
